# Seroprevalance of Brucellosis and Q-fever in water buffaloes (*Bubalus bubalis*) in Haryana state, India

**DOI:** 10.1371/journal.pone.0314726

**Published:** 2024-12-05

**Authors:** Dharanya Muthiah, Mahavir Singh, Rajesh Chhabra, Renu Gupta, Swati Dahiya, Priya, Jinu Manoj

**Affiliations:** 1 College Central Laboratory, COVS, Lala Lajpat Rai University of Veterinary and Animal Sciences, Hisar, Haryana, India; 2 Department of Veterinary Public Health and Epidemiology, COVS, Lala Lajpat Rai University of Veterinary and Animal Sciences, Hisar, Haryana, India; 3 Department of Veterinary Microbiology, COVS, Lala Lajpat Rai University of Veterinary and Animal Sciences, Hisar, Haryana, India; East Carolina University Brody School of Medicine, UNITED STATES OF AMERICA

## Abstract

Brucellosis and Q-fever are two highly contagious bacterial diseases with significant zoonotic potential and economic threats, yet they often remain underreported and neglected in low- and middle-income countries. The present study aimed to determine the seroprevalence of Brucellosis and Q-fever in water buffaloes in the Haryana state of India to implement effective preventive measures for disease control. The study covered all 22 districts of Haryana and involved 400 serum samples collected from female buffaloes belonging to two age groups and three distinct agro-climatic zones. The collected sera were tested using the Rose Bengal Plate agglutination test (RBPT) and commercial indirect enzyme linked immunosorbent assay (i-ELISA) for the presence of antibodies against smooth strains of *Brucella* spp. Additionally, the same serum samples were examined by i-ELISA for antibodies against *Coxiella burnetii*. The overall seroprevalence of brucellosis was observed as 8.25% (CI: 5.75–11.39) using the RBPT and 7.5% (CI: 5.12–10.53) by i-ELISA. The overall observed seroprevalence of Q-fever was 2.00% (CI: 0.87–3.90). No significant variation was seen in seropositivity of both diseases based on age and agroclimatic zones of the state. The findings of this study provide critical insights to farmers, agricultural organizations, veterinary services, and healthcare providers, facilitating more effective implementation of disease control measures.

## Introduction

India holds an extensive livestock population of 535.78 million heads, representing a 4.6% increase compared to the previous livestock census conducted in 2012 [1]. Among this diverse population, buffaloes hold a prominent position, constituting approximately 20.5% of the overall livestock numbers [1]. Haryana, the northern state of India stands as the native region for the renowned Murrah buffaloes, distinguished for their remarkable milking prowess and often referred to as "black gold". Buffaloes have emerged as the lifeblood of the farming community in Haryana. However, reproductive challenges such as abortions, infertility, and endometritis cast a shadow over the reproductive and milk production potential of dairy buffaloes and the farmer’s economy.

In domestic ruminants, brucellosis is one of the major causes of late-term abortion [2, 3]. The first on the list of zoonotic bacterial infections globally, brucellosis poses a serious threat to both animal and human health [4, 5]. In India, the economic losses based on brucellosis seroprevalence reports are in the tune of US$ 3.4 billion in livestock [6].

In animals, brucellosis frequently results in abortion storms, retained placenta, and endometritis in the infected herds and reduces the dairy animal’s ability to produce and reproduce [7]. Placenta, fetal fluids, vaginal discharges, and milk of infected animals are the main sources of transmission of brucellosis [8]. Humans may become infected by ingesting, inhaling, or coming into direct contact with these contaminated materials [3]. The disease manifests as undulant fever, joint pain, night sweats, and weakness in human beings. Prolonged exposure to the pathogen can lead to severe complications [9].

Parallel to the risk of brucellosis, Q-fever stands out as a significant zoonotic bacterial disease in livestock that inflicts reproductive and economic losses. It is caused by the Gram-negative bacterium *Coxiella burnetii* and affects a diverse range of mammals, including human beings, arthropods and birds [10]. Domestic ruminants (cattle, buffalo, sheep, and goat) are considered key reservoirs for *C*. *burnetii* [11]. Ingestion or inhalation of contaminated fetal fluids, uterine discharges and dust particles are the major routes of transmission to other in-contact animals of the herd. Furthermore, ticks may have a significant role in spreading the disease through biting as arthropod vectors [12]. The disease in buffaloes is mainly asymptomatic, however, late term abortions, stillbirths and other reproductive disorders including metritis and infertility [13] contribute to decreased productivity which in turn negatively affects the economy of the farmer. In human beings, Q-fever typically exists in asymptomatic, acute, and chronic forms. Acute cases display flu-like symptoms, hepatitis, and pneumonia, while the chronic form may manifest as chronic fatigue syndrome, endocarditis, or neurological issues. Additionally, Q-fever can result in abortions, stillbirths, and premature deliveries in pregnant women [13, 14].

Serological tests like the Rose Bengal plate agglutination test (RBPT), indirect enzyme linked immunosorbent assay (i-ELISA) and Complement fixation test (CFT) are recommended tests to study the herd prevalence of brucellosis in animals [15]. CFT is technically demanding and complex to perform as it involves titration of number of reagents and controls [16]. Serum agglutination test (SAT) is generally considered inadequate in animals for international trade purpose [15]. Fluorescence polarization assay (FPA) and competitive ELISA have the capability for differentiation of infected animals from vaccinated ones [16]. FPA is a rapid homogenous assay which does not involve repetitive washing steps for the separation of unbound analytes from the tube. Serological tests like i-ELISA, Immunofluorescence assay (IFA) and CFT are used for the diagnosis of Q-fever [13]. ELISA is a recommended test to carry out the seroprevalence study of Q-fever in animals [17].

There are not many systematic seroprevalence studies reported on brucellosis and Q-fever in female buffaloes in the state of Haryana, despite the huge population of buffaloes in the state. Female buffaloes account for approx. 90% of the total buffalo population in Haryana and mainly reared for milk production [1]. While the state is implementing the Brucellosis Control Program under the National Animal Disease Control Programme, aiming to provide 100% vaccination coverage to 4 to 8 months old female calves with the S19 calfhood vaccine, it’s still important to conduct seroprevalence studies to measure the presence of disease in the region [18]. Monitoring changes in seroprevalence over time is essential for assessing the effectiveness of preventive measures and understanding the impact of interventions on disease prevalence. Therefore, the goal of the current investigation was to screen representative samples from female buffaloes in Haryana to assess the seroprevalence of brucellosis and Q-fever. These studies aid in comprehending the scope and distribution of the disease and may be useful to dairy organizations, farmers, veterinary and human health services, and policymakers in developing successful local and national control and eradication plans.

## Materials and methods

### Ethics statement

This study was approved by the Institutional Animal Ethics Committee (IAEC), LUVAS, Hisar (Protocol No—IAEC/LUVAS/24/9).

### Study area

The study was conducted in all 22 districts of Haryana, India. It is located in northern India between 27°39’ to 30°35’ N latitude and between 74°28’ and 77°36’ E longitude, covering approximately 4.4 million hectares of land, which constitutes about 1.34% of the total geographical area of the country. The state is demarcated into three distinct zones (I, II and III), according to the ICAR-Agricultural Technology Application Research Institute, Jodhpur, based on their ecology and cropping patterns. Zone-I comprises eight districts, namely Panchkula, Ambala, Kurukshetra, Yamunanagar, Karnal, Kaithal, Panipat, and Sonipat, covering nearly 32% of the total area of the state. Zone-II consists of seven districts, namely Sirsa, Fatehabad, Hisar, Jind, Rohtak, Faridabad, and Palwal, accounting for nearly 39% of the total area. The regions falling under Zones I and II are conducive to crop diversification, offering favorable conditions for cultivating wheat, rice, pulses, cotton, and sugarcane, as well as for raising dairy cows, buffaloes, and poultry. These zones are characterized by better irrigation facilities and robust overall infrastructure. Zone-III includes seven districts, namely Bhiwani, Charkhi Dadri, Mahendergarh, Rewari, Jhajjar, Gurugram, and Mewat, covering nearly 29% of the total area. This zone primarily comprises areas devoted to pearl millet, rapeseed, and mustard cultivation, with suitability for arid horticulture as well [19].

### Study design

The study was conducted in 2022 and aimed to assess the seroprevalence of Brucellosis and Q-fever in female buffaloes of Haryana. The sample size was determined using the following equation, considering an expected prevalence of 50%, 95% confidence interval, and a desired absolute precision of 5% [20].


$$\text{n}=\frac{1.96^{2}\text{x}\;\text{P}\;\text{x}\left(1-\text{P}\right)}{{\text{d}}^{2}}$$

where P—expected prevalence

d—desired absolute precision

This calculation yielded a minimum requirement of 385 samples, rounded up to 400 samples. Serum samples were collected from the field as well as from the serum bank of the Department of Veterinary Microbiology, LUVAS, Hisar, Haryana, India. Serum samples sourced from serum bank used in this study were collected during the year 2022.

Samples were collected from female buffaloes falling into two age categories: 16–24 months and over 24 months. The first group comprised young stock buffaloes (n = 133), while the second group consisted of heifers, pregnant, and lactating buffaloes (n = 267). This categorization was based on data from the Haryana buffalo population, reflecting a ratio of young to adult buffaloes of 1:2 [1]. Additionally, female buffaloes above 16 months of age were included, as they typically attain puberty between the ages of 15–18 months, rendering them susceptible to brucellosis [21].

**Fig 1:**
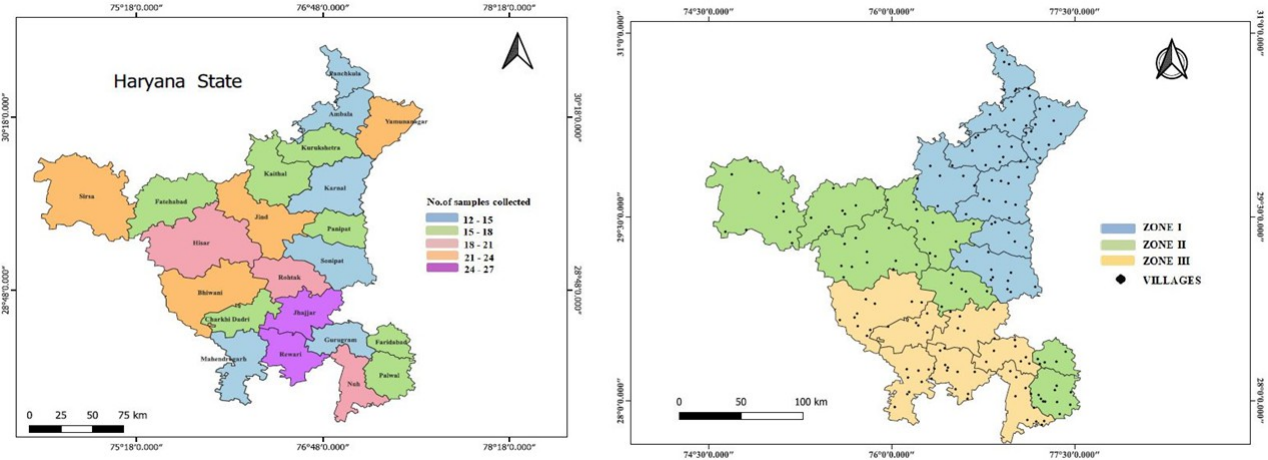
Geographical locations of sample collection sites in Haryana. (Left side) State (Haryana) map showing the number of samples collected from each district, (Right side) State map showing the individual sampling locations in different zones of Haryana.

Studies have indicated that the prevalence of Brucellosis and Q-fever varies according to agro-climatic zones due to the differences in climate, livestock population, land cover, cropping pattern, livestock farming practices and awareness among the farmers regarding these diseases across the zones [22–24]. Hence, the 400 samples collected were sourced from three distinct agro-climatic zones within Haryana to understand the transmission dynamics of the disease. Specifically, a total of 127, 137, and 136 serum samples were obtained from the female buffaloes belonging to zones I, II, and III of Haryana, respectively. To visualize the distribution of the study area, a map was created using QGIS version 3.22.12 (Fig 1).

### Sample preparation

Five ml blood sample was collected in a blood collection tube from the jugular vein of each animal after proper restraining. The blood collection tube was kept undisturbed in a slanting position at room temperature for 30–60 minutes. Samples were transported to the laboratory on ice packs. Blood samples were centrifuged at 2,500 x g for 10 minutes at room temperature. The serum was separated and pipetted into a labeled tube and stored at −20°C until further analysis.

### Serological screening of serum samples for brucellosis

#### Rose Bengal Plate test (RBPT)

All 400 serum samples of buffaloes were initially examined by RBPT. Antigen was procured from the Biological Products Division, ICAR-IVRI, Izatnagar, U.P., India. An equal volume (30 μl) of *Brucella* colored antigen and test serum were placed on a glass slide and mixed thoroughly for four minutes. Samples with any degree of agglutination were classified as positive, whereas those without any agglutination were considered negative.

#### Indirect enzyme linked immunosorbent assay

A commercial i-ELISA (IDEXX^®^ Brucellosis Serum X2, cat no. BAT1132T) procured from IDEXX Laboratories, USA, was used to screen the sera for antibodies against smooth strains of *Brucella* spp. ELISA protocol was used as per the manufacturer’s recommendations without any modification. Briefly, serum samples, positive and negative controls were dispensed (100μl) in appropriate wells of antigen coated microtitre plate. Then the plate was incubated at 37ºC for 60 minutes. Conjugate (100μl) was added in all the wells after three times washing of wells with 1x wash buffer. The plate was again incubated at 37ºC for 60 minutes. TMB substrate was added after three times washing of wells. The plate was again incubated at 18-26ºC for 15 minutes; after that reaction was stopped with a stop solution. Optical density values were measured at 450 nm using an ELISA reader (Synergy 2 microplate reader, Biotek, USA). The percent value of the sample to positive control (S/P%) was calculated for samples as per the manufacturer’s guidelines. S/P% values above 80 were recorded positive, while numbers below 80 were considered negative as per manufacturer’s guidelines.

$$\text{S}/\text{P}\%=100\;\text{x}\frac{\text{Sample}\;\text{A}\left(450\right)-\text{NC}\bar{\text{x}}}{\text{PC}\bar{\text{x}}-\text{NC}\bar{\text{x}}}$$

Where Sample A(450) = Sample optical density; $\text{PC}\bar{\text{x}}$ = mean value of positive control optical density; $\text{NC}\bar{\text{x}}$ = mean value of negative control optical density.

### Serological screening of serum samples for Q-fever

A commercial i-ELISA (IDEXX Q-fever, cat no. QFT1135T) procured from IDEXX Laboratories, USA, was used to screen the sera for antibodies against *C*. *burnetii*. ELISA protocol was used as per the manufacturer’s recommendations without any modification. Briefly, serum samples, positive and negative controls were prediluted 1/400 using 1x wash solution. Prediluted serum samples, positive and negative controls were dispensed (100μl) in appropriate wells of antigen coated microtitre plate. Then, the plate was incubated at 37ºC for 60 minutes. Conjugate (100μl) was added in all the wells after three times washing of wells. The plate was again incubated at 37ºC for 60 minutes. TMB substrate was added after three times washing of wells. The plate was incubated at 18-26ºC for 15 minutes; after that reaction was stopped with a stop solution. Optical density values were measured at 450nm using an ELISA reader (Synergy 2 microplate reader, Biotek, USA). The S/P% value of tested samples was calculated as per the manufacturer’s guidelines. S/P% values above 40 were recorded positive, while numbers below 30 were considered negative and values between 30 and 40 were considered suspect as per manufacturer’s guidelines.

$$\text{S}/\text{P}\%=100\;\text{x}\frac{\text{Sample}\;\text{A}\left(450\right)-\text{NC}\bar{\text{x}}}{\text{PC}\bar{\text{x}}-\text{NC}\bar{\text{x}}}$$

Where Sample A(450) = Sample Optical Density; $\text{PC}\bar{\text{x}}$ = mean value of positive control optical density; $\text{NC}\bar{\text{x}}$ = mean value of negative control optical density.

### Statistical analysis

The data was analysed using STATA^™^ (StataCorp, College Station, TX) software. For different agroclimatic zones and age groups, 95% confidence intervals of seropositivity were estimated. Pearson’s chi square/ Fisher’s exact test was applied to decipher the association between Brucellosis/ Q-fever seropositivity and agroclimatic zone and age of animals.

## Results

### Seroprevalance of Brucellosis in buffaloes

#### Rose Bengal plate test

In this study, a total of 400 serum samples were subjected to RBPT, revealing an overall seroprevalence of brucellosis as 8.25% (33/400). Among the various geographic zones, Zone II exhibited the highest seropositivity at 8.76% (12/137), closely followed by Zone III—8.09% (11/136) and Zone I—7.87% (10/127) (Table 1). However, statistical analysis using the Pearson chi-square test revealed no significant difference (P>0.05) between these zones. Further analysis with respect to age groups of female buffaloes revealed higher seropositivity of 8.27% (11/133) in buffaloes aged 16–24 months than in those above 24 months (8.24%; 22/267), with no statistical significant difference (P>0.05) between animals of the two age groups (Table 1).

**Table 1:** Association of different variables with the seropositivity of Brucellosis and Q-fever in buffaloes of Haryana.

Variables	Brucellosis							Q-fever*		
	No. of samples screened	Samples positive by RBPT (%)	95% CI	χ^2^ (p value)	Samples positive by iELISA (%)	95% CI	χ^2^ (p value)	Samples positive by iELISA (%)	95% CI	p value
**Zones**										
Zone I	127	10 (7.87)	3.84–14.00	0.0753^NS^	9 (7.09)	3.29–13.03	0.0908^NS^	1 (0.79)	0.02–4.31	0.547^NS^
Zone II	137	12 (8.76)	4.61–14.80	(0.963)	11 (8.03)	4.08–13.91	(0.956)	4 (2.92)	0.80–7.31	
Zone III	136	11 (8.09)	4.11–14.01		10 (7.35)	3.58–13.11		3 (2.21)	0.46–6.31	
**Age group**										
16–24 months	133	11 (8.27)	4.20–14.32	0.0001^NS^	9 (6.77)	3.14–12.46	0.1543^NS^	5 (3.76)	1.23–8.56	0.123^NS^
Above 24 months	267	22 (8.24)	5.24–12.21	(0.992)	21 (7.87)	4.93–11.77	(0.694)	3 (1.12)	0.23–3.25	

* Fisher’s exact test was applied.

RBPT- Rose Bengal Plate Agglutination Test, iELISA—Indirect Enzyme Linked Immunosorbent Assay, CI- Confidence Interval, %- percentage, χ^2^ –Chi square value, NS—Non Significant (p>0.05)

Among female buffaloes aged 16–24 months, Zone I recorded the highest seropositivity at 11.76% (6/51), followed by Zone III—7.32% (3/41) and Zone II—4.88% (2/41), with no statistical differences (P>0.05) detected across the zones. Similarly, for female buffaloes above 24 months of age, Zone II had the highest seropositivity at 10.42% (10/96) followed by Zone III—8.42% (8/95) and Zone I—5.26% (4/76) with no significant differences (P>0.05) between the zones (Table 2).

**Table 2:** Association of age with the seropositivity of Brucellosis and Q-fever in buffaloes of different zones of Haryana by fisher’s exact test.

Variables	Brucellosis							Q-fever		
	No. of samples tested	Samples positive by RBPT (%)	95% CI	p value	Samples positive by iELISA (%)	95% CI	p value	Samples positive by iELISA (%)	95% CI	p value
**16–24 months of age**										
Zone I	51	6 (11.76)	4.44–23.87	0.538^NS^	5 (9.80)	3.26–21.41	0.390^NS^	1 (1.96)	0.05–10.45	0.518^NS^
Zone II	41	2 (4.88)	0.60–16.53		1 (2.44)	0.06–12.86		3 (7.32)	1.54–19.92	
Zone III	41	3 (7.32)	1.54–19.92		3 (7.32)	1.54–12.46		1 (2.44)	0.06–12.86	
**Above 24 months of age**										
Zone I	76	4 (5.26)	1.45–12.93	0.500^NS^	4 (5.26)	1.45–12.93	0.489^NS^	0 (0)	0.00–4.74	0.641^NS^
Zone II	96	10 (10.42)	5.11–18.32		10 (10.42)	5.11–18.32		1 (1.04)	0.03–5.67	
Zone III	95	8 (8.42)	3.71–15.92		7 (7.37)	3.01–14.59		2(2.11)	0.26–7.40	

RBPT- Rose Bengal Plate Agglutination Test, iELISA—Indirect Enzyme Linked Immunosorbent Assay, CI- Confidence Interval, %- percentage, NS—Non Significant (p>0.05)

### Indirect enzyme linked immunosorbent assay

All the 400 serum samples were analyzed through i-ELISA, showing an overall seroprevalence of 7.5% (30/400). Among different zones, Zone II showed the highest seropositivity of 8.03% (11/137), followed by Zone III- 7.35% (10/136) and Zone I- 7.09% (9/127). Despite these variations, statistical analysis revealed no significant difference (P>0.05) among these zones. Further examination by age group showed brucellosis positivity of 6.77% (9/133) in female buffaloes aged 16–24 months and 7.87% (21/267) in those above 24 months. However, there were no statistical significant differences (P>0.05) detected between these age groups (Table 1).

Zone-wise analysis of seropositivity in female buffaloes aged 16–24 months and above 24 months revealed specific patterns, with no significant differences (P>0.05) observed among the zones (Table 2). Notably, Zone I exhibited the highest seropositivity of 9.8% (5/51) in younger buffaloes and the lowest at 5.26% (4/76) in older buffaloes, while Zone II showed the highest seropositivity of 10.42% (10/96) in older buffaloes (Table 2).

### Seroprevalence of Q-fever in buffaloes

In our investigation, 2% (8/400) of serum samples tested by i-ELISA were found positive for the presence of antibodies against *C*. *burnetii*. Among different zones, Zone II showed the highest seropositivity of 2.92% (4/137), followed by Zone III—2.21% (3/136) and Zone I—0.79% (1/127) with no significant difference (P>0.05) among zones. Seropositivity of Q-fever in female buffaloes aged 16–24 months was 3.76% (5/133) and 1.12% (3/267) in those above 24 months, showing no significant differences(P>0.05) among these age groups (Table 1).

Analyzing female buffaloes aged 16–24 months across zones revealed the highest seropositivity in Zone II—7.32% (3/41) followed by Zone III—2.44% (1/41) and Zone I—1.96% (1/51), with no statistically significant difference (P>0.05) within these zones. For female buffaloes above 24 months, Zone III had the highest seropositivity of 2.11% (2/95) followed by Zone II—1.04% (1/96) and no seropositivity reported in Zone I (0/76) and no significant difference (P>0.05) among these zones (Table 2).

## Discussion

Brucellosis and Q-fever are zoonotic diseases that affect the reproductive potential of dairy animals [2, 3, 13]. There may also be a possibility of co-infection of Q-fever and brucellosis in ruminants as well as in human beings which may exaggerate the pathogenesis and impact of these zoonotic diseases [2, 25]. Abortion after five months of gestation in cattle and buffaloes should be properly investigated for the presence of infectious agents in animals [15]. Brucellosis is widely distributed in many countries around the world [4]. The majority of late term abortions in buffaloes are due to brucellosis in India [3]. The disease is usually diagnosed through serological techniques such as RBPT, SAT, and ELISA, as well as culture methods. While the isolation of *Brucella* spp. via culture is considered the gold standard, but its practicality is limited due to the requirement for BSL-3 facilities and heightened zoonotic risks [26]. Serology offers a practical alternative to culture-based approaches for diagnosing brucellosis. RBPT appears to be adequate as a herd screening test due to its cost-effectiveness and quickness in providing results [15, 27]. ELISA is a widely used serological test for seroprevalence studies of brucellosis [28, 29].

In this study, 400 serum samples were tested utilizing both the RBPT and i-ELISA. Among these samples, 33 were identified as positive through RBPT, while 30 samples were positive by i-ELISA. This yielded a higher seroprevalence of brucellosis by RBPT (8.25%) than i-ELISA (7.50%). These two tests exhibited substantial agreement (k value = 0.7071). The results of earlier studies carried out in different states of India like in Gujarat (9.59%) [30], Tamil Nadu (6.66%) [31] and Rajasthan (9.66%) [32] were consistent with the RBPT based seroprevalence of brucellosis in buffaloes found in this study. However, only a few studies [33–37] have estimated lower frequency in buffaloes, ranging from 1.9 to 5.65% by RBPT. In contrast, a greater herd prevalence of brucellosis was also reported by various researchers ranging from 12.73% to 35.22% in India and Pakistan [38–40].

According to i-ELISA, seroprevalence of brucellosis was estimated 7.5%, which was comparable to previous estimates of seroprevalence in buffaloes as 5.98% [36] and 5.6% [27] in Punjab, Pakistan. At the global level, a study reported 9.7% seroprevalence of brucellosis in water buffaloes using a meta-analysis approach [41]. In Sub-Saharan Africa, a study reported 8–46% prevalence of brucellosis in domestic ruminants [42]. By using i-ELISA, several studies have shown that buffaloes had higher seroprevalence, ranging from 12.33% to 26.03% [34, 40, 43, 44]. Fewer studies reported a lower seroprevalence of 2.1% [35] in Andhra Pradesh, 2.66% in Rajasthan of India [37] and 2.87% [33] in Bangladesh. A meta-analysis study of 2018 suggested the overall prevalence of bovine brucellosis in India as 12% or less [45]. Another meta-analysis study of 2023 revealed 16.6% and 14.2% prevalence of brucellosis in cattle and buffaloes respectively in India [46]. Variation in seroprevalence reported by various researchers may be attributed to differences in sampling techniques, selection of animal farms, and management strategies employed in specific regions.

The highest seropositivity of brucellosis was observed in Zone II, followed by Zone III and Zone I. These variations in positivity across different zones can be attributed to differences in rearing practices, livestock population, and pasture areas. Zone II is characterized by abundant land cover, pasture areas and a higher density of livestock population, particularly buffaloes [1]. This environment may foster interactions between different species, thereby facilitating the transmission of diseases. Nonetheless, the observed seropositivity pattern of brucellosis in female buffaloes across the zones of Haryana did not demonstrate statistical significance (p > 0.05). This result aligns with the findings of a study [39] which similarly concluded that there was no significant difference in the frequency of the disease among different agro-climatic regions of Punjab, India.

The seropositivity of brucellosis appears to be higher in older age groups (>24 months of age) compared to younger animals (16–24 months of age). While brucellosis affects bovines across all age groups, sexually mature animals are often more susceptible to the disease [47]. On the other hand, the observed seropositivity in young animals in this study could be linked to factors such as consumption of milk from infected dams, prolonged exposure to highly contaminated farm environments, and potential in-utero transmission of the disease from the dam to the fetus. However, the differences in seropositivity among different age groups were not statistically significant. Similarly, some researchers [27, 48] reported statistically non-significant seropositivity results among different age groups.

Furthermore, the seropositivity of brucellosis in female buffaloes aged 16–24 months was higher in Zone I, whereas Zone II recorded a higher seropositivity in female buffaloes above 24 months of age. However, there was no statistical significance in seropositivity among different age groups across various agro-climatic zones. This observation could be attributed to common farm management practices implemented in the study area, including rigorous hygiene and quarantine procedures, appropriate disposal methods for placenta/aborted fetuses, and other effective animal husbandry practices.

Q-fever, another bacterial disease with reproductive and economic losses in livestock, is categorized as one of the “13 global zoonoses” [49]. ELISA stands as the preferred technique for serological monitoring of Q-fever in animals, owing to its higher sensitivity and specificity [50]. In the present study, the overall seroprevalence of Q-fever was estimated as 2% in the buffaloes of Haryana. Our findings align with a study [51], which reported a prevalence of 3.9% in Punjab, India. However, other researchers have documented higher prevalence of disease in buffaloes ranging from 8.3% to 28% [13, 24, 49]. In India, seroprevalence of Q-fever in bovines (cattle and buffaloes) was reported 5.4% [52], 6.3% in cattle [51] and 17.7% in cattle [49]. In other parts of the world, the seroprevalence of Q-fever in cattle was reported 8.13% in Nandi County, Kenya [53], 24.28% in Limpopo province, South Africa [54] and 20.5% in Guinea [55].

In our study, we noted differing levels of seropositivity across different geographical zones. Specifically, Zone II displayed the highest seropositivity, whereas Zone I exhibited the lowest. The dense livestock population in Zone II may facilitate environmental contamination [55, 56] and increase the risk of dissemination and transmission of *C*. *burnetii* to neighboring areas through airborne particles [57].

The seropositivity was found to be higher in younger animals (16–24 months of age) compared to older animals (>24 months of age). Furthermore, the seropositivity of Q-fever in female buffaloes aged 16–24 months was higher in Zone II, whereas Zone III recorded a higher seropositivity of Q-fever in female buffaloes above 24 months of age. Young animals might have acquired infection through contact with infected animals, exposure to contaminated environments, or ingestion of infected mother’s milk [58]. Additionally, a study [59] reported an association between ticks and a high prevalence of Q-fever.

The higher seropositivity observed in above 24 months aged buffaloes of Zone III may be attributed to the increased probability of exposure to the pathogen in adult animals [60]. Additionally, animals after their first calving are reported to be more susceptible to *Coxiella* infection, potentially contributing to the higher seropositivity in this age group [61]. Nevertheless, statistical analysis did not reveal any significant association among different zones or age groups within the zones of Haryana.

In conclusion, the present study revealed a seroprevalence of 8.25% for brucellosis in buffaloes using RBPT and 7.50% using i-ELISA in Haryana, India. The study revealed seroprevalence of 2% for Q-fever in buffaloes of Haryana, India. In addition, only one sample of buffalo was found serologically positive for both brucellosis and Q fever. Moreover, the study’s comprehensive approach, covering all the districts of the state, contributes to a better understanding of the disease distribution and prevalence within the state.

## Supporting information

S1 Data (XLSX)
